# Evaluating the Efficiency of DNA Metabarcoding to Analyze the Diet of *Hippocampus guttulatus* (Teleostea: Syngnathidae)

**DOI:** 10.3390/life11100998

**Published:** 2021-09-22

**Authors:** Tamara Lazic, Cataldo Pierri, Giuseppe Corriero, Bachir Balech, Frine Cardone, Michele Deflorio, Bruno Fosso, Carmela Gissi, Marinella Marzano, Francesco Nonnis Marzano, Graziano Pesole, Monica Santamaria, Michele Gristina

**Affiliations:** 1 Department of Biology, University of Bari, 70125 Bari, Italy; tamara.lazic@uniba.it (T.L.); giuseppe.corriero@uniba.it (G.C.); 2 Institute of Biomembrane, Bioenergetics and Molecular Biotechnology (IBIOM), National Council of Research (CNR), 70121 Bari, Italy; b.balech@ibiom.cnr.it (B.B.); b.fosso@ibiom.cnr.it (B.F.); carmela.gissi@uniba.it (C.G.); m.marzano@ibiom.cnr.it (M.M.); graziano.pesole@uniba.it (G.P.); m.santamaria@ibiom.cnr.it (M.S.); 3 Department of Integrated Marine Ecology, Zoological Station Anton Dohrn, 80127 Naples, Italy; frine.cardone@szn.it; 4 Department of Veterinary Medicine, University of Bari, 70125 Bari, Italy; michele.deflorio@uniba.it; 5 Department of Biosciences, Biotechnology and Biopharmaceutics, University of Bari, 70125 Bari, Italy; 6 Department of Chemistry, Life Sciences and Environmental Sustainability, University of Parma, 43121 Parma, Italy; francesco.nonnismarzano@unipr.it; 7 Institute of Anthropic Impacts and Sustainability in Marine Environment (IAS), National Council of Research (CNR), 90100 Palermo, Italy; michele.gristina@cnr.it

**Keywords:** seahorses, *Hippocampus guttulatus*, diet, DNA metabarcoding

## Abstract

Seahorses are considered a flagship species for conservation efforts and due to their conservation status, improving knowledge on their dietary composition while applying a non-invasive approach, could be useful. Using *Hippocampus guttulatus* as a case study, the present study represents pioneering research into investigating the diet of seahorses by NGS-based DNA metabarcoding of fecal samples. The study developed and tested the protocol for fecal DNA metabarcoding during the feeding trials where captive seahorses were fed on a diet of known composition; the process was subsequently applied on fecal samples collected from wild individuals. The analysis of samples collected during the feeding trials indicated the reliability of the applied molecular approach by allowing the characterization of the effectively ingested prey. In the field study, among detected prey species, results revealed that the majority of the seahorse samples contained taxa such as Amphipoda, Decapoda, Isopoda, and Calanoida, while less common prey taxa were Gastropoda and Polyplacophora. As only a small amount of starting fecal material is needed and the sampling procedure is neither invasive nor lethal. The present study indicates DNA metabarcoding as useful for investigating seahorse diet and could help define management and conservation actions.

## 1. Introduction

The knowledge of a species’ dietary composition is a keystone to understanding the way it exploits the environment and to designing effective management and conservation plans [1]. Indeed, development of the accurate methods to study the diet is an active area of research and attracts growing interest in conservation studies [2].

Dietary composition is traditionally determined through visual recognition of morphological features in samples of stomach, gut, or fecal contents using macro- and microscopic methods [3]. While useful, such identifications rely on considerable taxonomic expertise to classify portions of undigested prey [4]. Some potential prey species, such as crustaceans, could be morphologically similar to one another [5], making the process taxonomically challenging [6]. Furthermore, soft-bodied taxa, such as nematodes and polychaetes, digest in a short period of time (1–3 h) leaving no visual remains [7,8]. As such, traditional analysis of fecal samples can result in a considerable information loss and poor resolution of determining taxa, while sometimes requiring the sacrifice of animals [9].

In recent years, remarkable progress has been made towards developing accurate, non-invasive, and cost-efficient alternatives. As a result, DNA amplicon-based analyses of fecal samples, also referred as fecal DNA metabarcoding, has been adopted and become a valuable tool for ecological research [10]. Based on Next Generation Sequencing (NGS), it allows the analysis of taxon-specific variants of standardized genomic regions, i.e., DNA barcodes, which are amplified from DNA mixtures by universal PCR primers. This approach, therefore, virtually enables the identification of all organisms contained in a sample [2,11]. Although it cannot provide information on prey size or life cycle stage nor it is immune to prey taxa retention due to differential digestion and gut transition times, DNA metabarcoding perpetuates non-invasiveness, offers high taxonomic resolution, and has high sensitivity towards soft, highly degraded, and rare prey species [12,13,14]. Since its inception, DNA metabarcoding has been successfully applied in dietary studies of many species, including fish, and provided encouraging results [12,15,16].

Seahorses (*Hippocampus* spp.) are small predatory fish, considered a “flagship” species for conservation efforts [17]. In the past decades, severe decline of many seahorse populations caused numerous concerns and led to the inclusion of all seahorse species, including the long-snouted seahorse *Hippocampus guttulatus*, on the International Union for Conservation of Nature (IUCN) Red List of Threatened Species [18] and in Appendix II of the Convention on International Trade in Endangered Species of Wild Fauna and Flora [19].

Seahorses usually practice a “sit-and-wait” predation strategy when they wait for prey from a hidden position and then launch a rapid surprise attack [20]. By employing morphological examination of gut or stomach contents, through stomach flushing or by biochemical means, studies on their diet have shown that seahorses feed mainly on small-sized crustaceans, such as Amphipoda, Decapoda, and Anomura [20,21,22,23]. A recent study based on morphological examination of stomach contents revealed the presence of only a few soft-bodied prey items, such as nematodes [20]. Indeed, traditional diet methods tend to underestimate the frequency of occurrence of prey items with totally digested body parts, such as soft-bodied arthropods [24], while these methods tend to more easily detect hard-bodied groups [25]. Although it has been shown that the prey’s DNA is recoverable from seahorse feces [15], there have been no published studies demonstrating the use of highly sensitive and non-invasive molecular techniques to evaluate the entire spectrum of seahorses’ dietary items despite the sensitive conservation status.

Using *H. guttulatus* as a case study, the purpose of this research was to validate the potential of DNA metabarcoding approach to globally identify prey fecal content while developing an effective and reliable protocol that could be further applied in wild populations. Importantly, as *H. guttulatus* is considered a Near Threatened (NT) species in both the Mediterranean Sea and along the Italian coast [18], it is of value to develop a non-lethal and non-invasive method to study the species diet. By maximizing prey detectability and identifying optimal designs for field studies, it has been shown that feeding trials with captive animals and known diets are crucial [26]. Therefore, the present study included feeding trials under controlled laboratory conditions in which captive seahorses were fed on a diet of known composition and the developed protocol was subsequently applied on fecal samples collected from wild individuals.

## 2. Material and Methods

### 2.1. Feeding Trials

Feeding trials were performed with four non-reproductive *H. guttulatus* female specimens collected by diving at Taranto Mar Piccolo (Ionian Sea 40°28′ N, 17°16′ W; for more details see [27]). Specimens were transported to the facilities of agricultural society “Ittica Caldoli S.r.L.” and maintained in individual 30 L aquaria. Seawater inside the aquaria was filtered through 0.2 μm pore-size polycarbonate filters. The seawater temperature was maintained at 18 ± 0.5 °C, salinity at 36 ± 1‰, pH at 8.0 ± 0.2, and the photoperiod was adapted to the natural day cycle. Three prey items (*Gammarus aequicauda*, *Palaemon elegans* and *Perinereis aibuhitensi*) were collected at Taranto Mar Piccolo in an area where seahorses are present and were fed with *Artemia* metanauplii and *Nannochloropsis oceanica*. The fourth prey item, adult *Artemia franciscana,* was cultured in the laboratory and fed with *N. oceanica.* All prey species were taxonomically identified under the microscope. Before the beginning of the experiment and between successive feeds with different prey species, seahorses were starved for 24 h to ensure an empty gut [15]. Seahorses were fed simultaneously on a single prey species added daily (at 09:00 am) at a single dose ad libitum, according to the following sequence: *G. aequicauda* at day 1, *A. franciscana* at day 3, *P. elegans* at day 5, and *P. aibuhitensis* at day 7. Uneaten prey was removed from the aquarium the day after experimental feeding (at 09:00 am). The feces (n = 10; three from the diet with *A. franciscana*, three from *P. elegans*, two from *G. aequicauda*, and two from *P. aibuhitensis*) produced in aquaria were immediately collected by syphoning. Samples of prey species and feces were preserved in 96% ethanol and stored at −20 °C for subsequent molecular analysis. At the end of the trials, all animals were released to the original capture site in perfect health conditions.

### 2.2. Field Study

In the field study, thirteen adult non-reproductive seahorses were captured by diving at Taranto Mar Piccolo at the same site as seahorses used in feeding trials. Animals were collected in two habitats: (1) *Corallina elongata* on vertical artificial substrates at 0.3–0.6 m of depth (n = 7) and (2) *Cladophora prolifera* at 6 m of depth (n = 6). Seahorses were individually placed in small aerated 2L tanks filled with surrounding water filtered through 0.2 μm pore-size polycarbonate filters. Nine fecal samples produced (three from seahorses found in *C. prolifera* and six in *C. elongata*) were collected by syphoning. After the fecal collection, all individuals were immediately released in a range of a maximum of 2 m from the capture spot. Fecal samples were preserved in 96% ethanol and stored at −20 °C for further examination. To exclude host contamination, skin filament tissue of *H. guttulatus* was sampled using skin filament clipping technique [28] and stored at the same conditions as fecal samples.

### 2.3. DNA Extraction

Total genomic DNA was extracted from fecal (50 mg), prey, and skin filament samples using FastDNA SPIN kit for soil (BIO 101, Carlsbad, Canada) following the manufacturer’s instructions. Cell lysis was achieved by bead beating in FastPrep Instrument (BIO 101) at speed 6 for 40 s. Qualitative and quantitative DNA assessment was carried out using PicoGreen^®^dsDNA quantitation assay (Invitrogen, Carlsbad, CA, USA) and agarose gel (1%) electrophoresis. DNA extraction blanks (sterile distilled water) were prepared and processed together with the samples to exclude any contamination related to the extraction reagents and procedure. DNA extracts were stored at −20 °C prior to amplification by PCR.

### 2.4. Cox1 Library Preparation and Sequencing

DNA metabarcoding approach was applied to the extracted DNA to identify eukaryotic diversity of fecal samples collected during both feeding trials and field study. The mitochondrial Cox1 gene was chosen as a molecular target because it: (i) represents one of the preferred loci for “universal” barcoding in Eukaryotes [29], (ii) has high resolution power due to its high variability between species [30], and (iii) has been already successfully used in previous dietary studies on fish [31,32].

Amplicon libraries were prepared from 0.5 ng of extracted DNA. The adopted strategy is described in detail in [33].

The adopted primer pair was mlCOIintF_NextFor and dgHCO2198_NextRev (Next_For: 5′-TCGTCGGCAGCGTCAGATGTGTATAAGAGACAG-3′, and Next_Rev: 5′GTCTCGTGGGCTCGGAGATGTGTATAAGAGACAG-3′) [32], designed to contain (from 5′ to 3′ ends) transposon Nextera sequences (Nextera DNA sample preparation guide, Illumina). RNase/Dnase-free Molecular Biology Grade water (Ambion) was used as a negative control of PCR amplification. Equimolar quantities of the purified amplicons were pooled and subjected to 2 × 250 bp paired-end sequencing on the Illumina MiSeq platform. To increase genetic diversity of the sequenced samples, as required by the MiSeq platform, a phage PhiX genomic DNA library was added to the mix and co-sequenced [34].

Simultaneously, DNA extracted from four preys and skin filament of *H. guttulatus* was amplified using primer pair mlCOIintF and dgHCO2198 [32]. The amplification was performed using Phusion^®^ High-Fidelity DNA polymerase (Thermo Fisher Scientific, Inc., New England Biolabs) in the Mastercycler Thermal Cycler (Eppendorf, Hamburg, Germany). Each reaction mixture contained 0.5 ng of extracted DNA, 5X Buffer HF, 10 mM dNTPs, 10 μM of each primer, and 1U Phusion DNA Polymerase in a final volume of 50 μL. The cycling parameters for PCR were standardized as follows: initial denaturation at 98 °C for 30 s, followed by 15 cycles of denaturation at 98 °C for 10 s, annealing at 54 °C for 30 s, extension at 72 °C for 15 s, and subsequently 20 cycles of denaturation at 98 °C for 10 s, annealing at 45 °C for 30 s, extension at 72 °C for 15 s, with the final extension step of 7 min at 72 °C. All PCRs were performed in the presence of a negative control (Rnase/Dnase-free Molecular Biology Grade water, Ambion). PCR products were visualized on 1.3% agarose gel and purified using AMPure XP Beads (Agencourt Bioscience Corporation, Beverly, Massachusetts) at a concentration of 0.8× vol/vol. PCR products were subjected to Sanger DNA sequencing by Eurofins Genomics (www.eurofinsgenomics.com (accessed on 1 September 2021)).

### 2.5. Taxonomic Analyses

The quality of raw Cox1 sequence data was checked using FastQC (Available online: http://www.bioinformatics.babraham.ac.uk/projects/fastqc/ (accessed on 1 September 2021)) and multiQC [35]. Illumina adapters and PCR primers were removed from raw reads using cutadapt [36]. Retained PE reads were denoised into ASVs (Amplicon Sequence Variants) [37] by applying DADA2 (version 1.10.1) [38]. ASVs were taxonomically annotated using a modified version of BioMaS (Bioinformatic analysis of Metagenomic amplicons) pipeline [39] working on MetaCOXI and MIDORI [40] as reference databases. Dynamic sequence similarity threshold was adopted to improve the ASVs classification accuracy (species at 97%, genus at 95%, family at 93%, class at 91%, order at 88%, and phylum at 78%) as in Lotus pipeline [41]. In particular, the taxonomic classification to a specific rank (e.g., family) was accepted exclusively if the matches obtained against the reference collection reached the imposed threshold. For example, taxonomic classification to the family rank was accepted only if the supporting matches reached at least 93% of similarity. Contaminant ASVs were identified using *decontam* [42] and aligned against *H. guttulatus* Cox1 sequence, human genome, and the release 138 of SILVA database [43] allowing the removal of noise. Unassigned ASVs were finally aligned against non-redundant blast nt collection (ftp.ncbi.nlm.nih.gov/blast/documents/blastdb.html (accessed on 1 September 2021)) using blast [44]. All ASV sequences partially mapping on different reference sequences were labelled as chimeric and removed from subsequent analysis. Retained sequences were taxonomically annotated using TANGO [45,46] and the same similarity percentage thresholds described above. Finally, unclassified ASVs were aligned against the non-redundant protein nr collection (ftp.ncbi.nlm.nih.gov/blast/documents/blastdb.html (accessed on 1 September 2021)) using blastx option of DIAMOND tool [47] and taxonomically classified with TANGO. R packages *phyloseq* (1.26.1) [48] and *vegan* (2.5.6) [49] were used to measure alpha and beta diversity. For this purpose, ASVs counts were initially filtered to remove low abundance sequences (total relative frequencies <10^−5^) and normalized by rarefaction (depth values settled to 123,000). Shannon and Simpson indexes were used as measures of alpha diversity (i.e., intra-sample diversity), while Bray–Curtis dissimilarity matrix was used to measure beta diversity (i.e., inter-sample diversity). Statistical differences in alpha diversity indexes were measured by Wilcoxon (W) tests. PERMANOVA (Permutational Multivariate analysis of variance) was measured to infer the contribution of explanatory variables in beta diversity data partitioning by applying 999 permutations. The contribution of individual species to the overall Bray–Curtis dissimilarity was achieved by SIMPER analysis with 999 permutations.

## 3. Results

### 3.1. Overall Sequencing Results

Libraries of dual indexed amplicons of 420 bp were successfully sequenced on the MiSeq platform using 2 × 250 bp paired-end (PE) sequencing strategy. Approximately 7.6 million PE reads (mean 401,702 ± 67,992 S.D.) were generated in two different sequencing runs. After noise and contaminant removal, 99.7% of the produced PE reads were retained (Figure S1), resulting in a total of 552 ASVs.

According to the ecological metrics, data were normalized by rarefaction to 123,000 sequences. One sample from the wild specimen was discarded as it contained less sequences (15,706) than the imposed rarefaction depth and was dominated by *H. guttulatus* Cox1 gene sequences.

### 3.2. Feeding Trials

The designed experimental protocol successfully detected all four prey items fed to the captive seahorses. A total of 241 ASVs were retained after contaminant and noise filtering and were taxonomically classified at least at kingdom level.

In the three fecal samples of animals fed on *A. franciscana*, the most represented species were *N. oceanica* and *A. franciscana* (Table 1).

In the two samples of animals fed on *G. aequicauda,* the most represented taxon was *Gammarus* sp. but none of the assigned AVSs was classified at species level, except *N. oceanica* (Table 2).

In the two fecal samples of animals fed on *P. aibuhitensis*, the most represented species were *N. oceanica* and *P. aibuhitensis*. The most abundant genus was *Urodasys* (Table 3).

In the three fecal samples collected from seahorses fed on *P. elegans*, the most abundant species were *P. elegans*, *Ophryotrocha labronica* and *N. oceanica*. *Alpheus bellulus* was also observed (Table 4).

**Figure 1:**
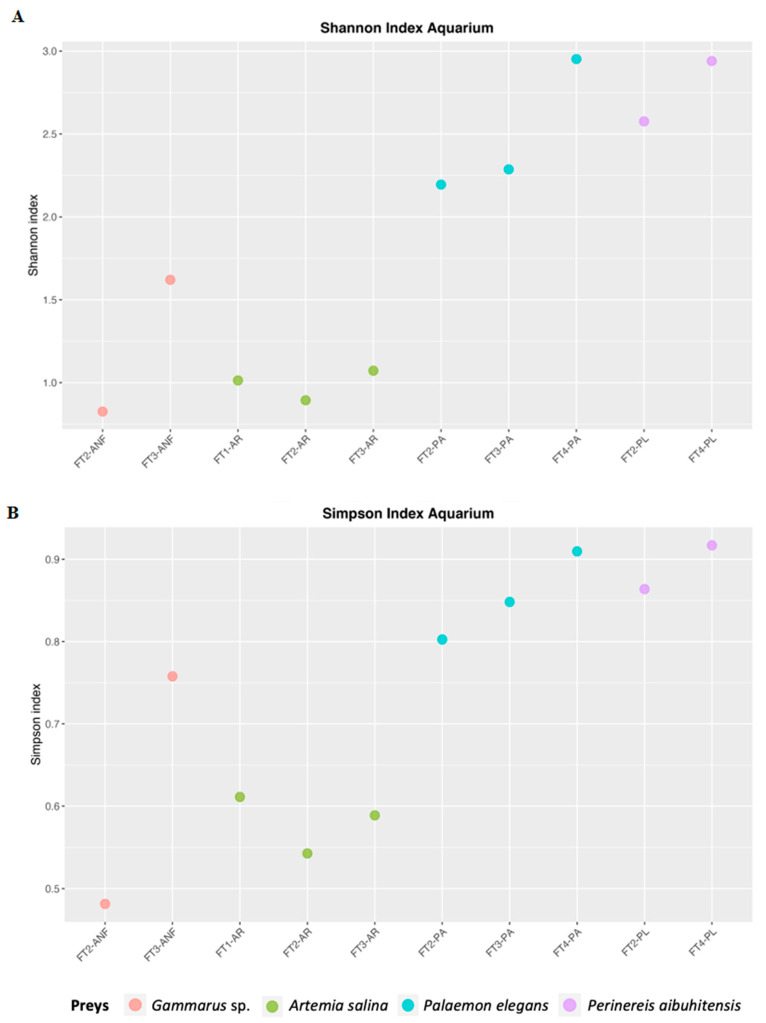
Shannon (**A**) and Simpson (**B**) indices of aquarium samples.

**Figure 2:**
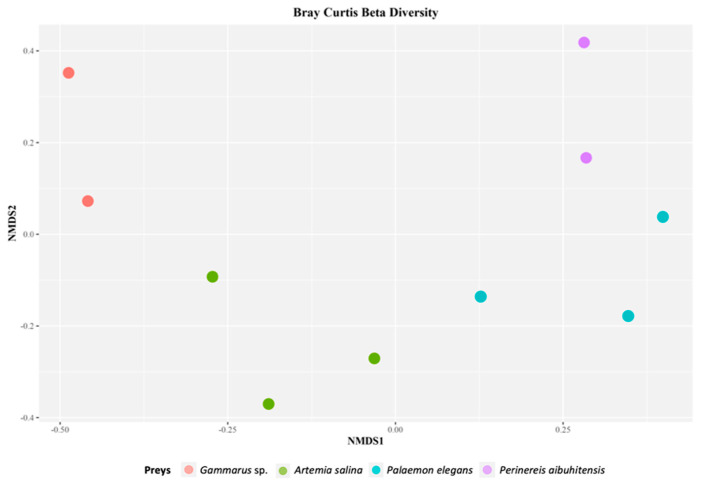
nMDS plot based on the Bray–Curtis dissimilarity matrix of aquarium samples.

Shannon and Inverted Simpson Indices (Figure 1) indicated a high variability in aquarium samples (0.85 ÷ 2.95, IQR = 1.47 and 0.48 ÷ 0.92, and IQR = 0.26). Beta diversity (Figure 2) indicated sample clustering according to the applied diet. According to the PERMANOVA model, the seahorse diet explains 67% of the observed variability (permutational *p*-value = 0.002).

The SIMPER analysis allowed us to identify the ASVs that significantly (999 permutations) contributed to the observed dissimilarities among seahorses fed with different prey (Table S1).

### 3.3. Field Study

A total of 282 ASVs were retained in samples after contaminant and noise filtering. All ASVs were taxonomically classified at least at the kingdom level.

Four classes (Malacostraca, Hexanauplia, Gastropoda, and Polyplacophora) were observed, representing six orders (Amphipoda, Calanoida, Isopoda, Decapoda, Neogastropoda, and Chitonida), and six families (Gammaridae, Acartiidae, Munnidae, Alpheidae, Muricidae, and Pseudodiaptomidae). At the genus level, several taxa were observed: *Munna* (Arthropoda, 16.5% ± 32.5%, in six out of nine samples), *Athanas* (Arthropoda, 14.29% ± 28.4%, in six samples), *Gammarus* (Arthropoda, 7.16% ± 21.46%, in four samples), *Paracartia* (Arthropoda, 10.93% ± 22.97%, in six samples), and *Urosalpinx* (Mollusca, 5.97% ± 17.8%, in three samples). At the species level, *Munna japonica* (Arthropoda, 16.5% ± 32.4%, in six samples out of nine), *Athanas nitescens* (Arthropoda 14.3% ± 28.4%, in six samples), *Paracartia grani* (Arthropoda 10.9% ± 23%, in two samples), and *Urosalpinx cinerea* (Mollusca 5.96% ± 17.9%, in two samples) were observed. The abundance of unassigned sequences at species level ranged between 10.32% and 99.81% (mean relative abundance 50.9 ± 40.79%).

**Figure 3:**
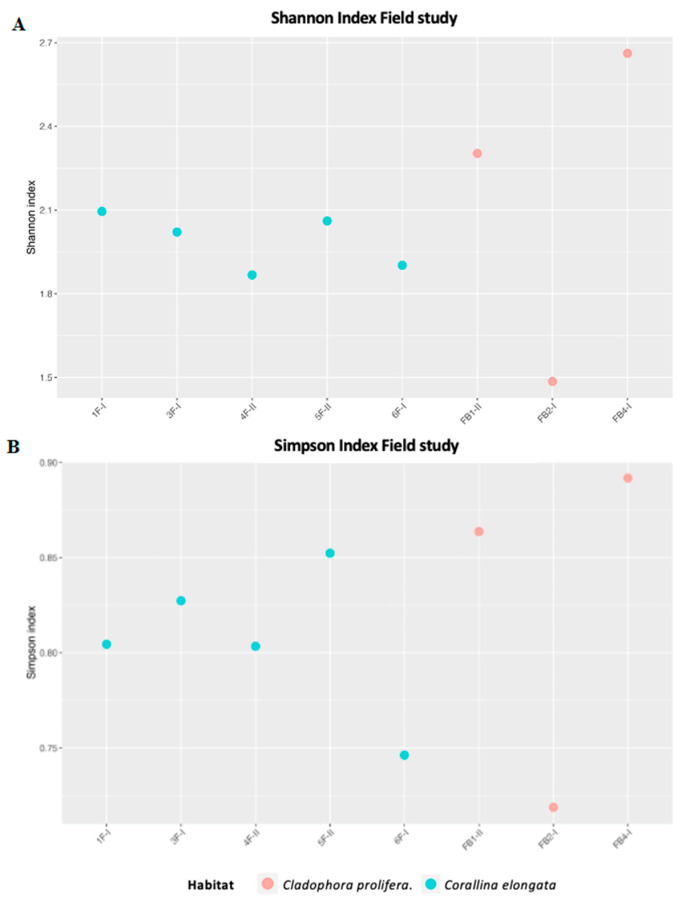
Shannon (**A**) and Simpson (**B**) indices of field samples.

Measured Shannon and Simpson indices (Figure 3) showed similar values in five samples out of eight (1.48 ÷ 2.66, IQR = 0.26 and 0.72 ÷ 0.89, and IQR = 0.07).

**Figure 4:**
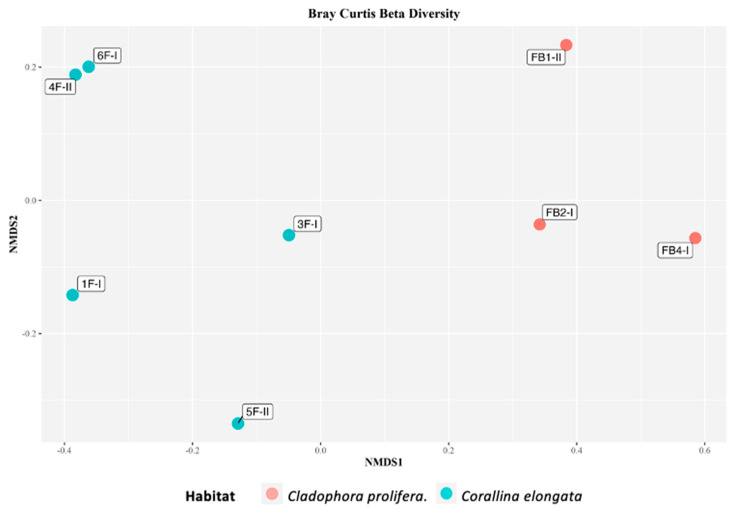
nMDS plot based on the Bray–Curtis dissimilarity matrix of field samples.

Beta diversity (Figure 4), measured by Bray–Curtis dissimilarity metrics and plotted using nMDS (non-metric Multi-Dimensional Scaling), indicated that the samples were mostly grouped according to the habitat from which seahorses were sampled.

## 4. Discussion

The present study represents pioneering research in investigating the diet of seahorses by NGS-based DNA metabarcoding while demonstrating that the applied approach is useful to provide information on their diet. Importantly, it proved to be efficient in obtaining dietary information with minimal disturbance for individuals. In the field study, among detected prey species, results revealed that the majority of the seahorse samples contained crustacean taxa such as Amphipoda, Decapoda, Isopoda, and Calanoida. The less common prey taxa were Gastropoda and Polyplacophora with only one detection across nine samples.

Observation of crustaceans as dominant prey is in congruence with previous studies, based on conventional morphological identification methods [20,21,22], although from a taxonomic point of view, the present results were more accurate and consistent in terms of the identified taxa. Despite the small number of feces analyzed (n = 9), the DNA metabarcoding approach corroborates and provides additional resolution to results from these studies. Dietary habits of wild long-snouted seahorses, assessed by DNA metabarcoding, encompassed several species that have, to our knowledge, not been identified previously as potential seahorse preys, namely *Munna japonica*, *Athanas nitescens*, *Paracartia grani grani*, and *Urosalpinx cinerea*. Moreover, the great majority of prey taxa were identified at the species level, while previous work at Taranto Mar Piccolo only provided identification at the order or family level [20]. The appearance of non-native species in the seahorse diet is particularly interesting. Seahorses seem to show an adaptive capacity in lagoons in which the presence of non-native species is the norm rather than the exception. *P. grani grani* is, indeed, a non-native species for the Italian seas. According to the literature, *Munna japonica* and *U. cinerea* have never been recorded along the Italian coast, and thus, should be considered as non-native. However, although both species were detected by DNA metabarcoding, their identification as non-native species requires more in-depth studies.

Feeding trials with captive animals and known diets allow the approach to be trialed while identifying optimal design for field studies [2] since prey DNA detectability can be influenced by different biological and technical factors that could considerably affect the diet inference from fecal DNA analysis [2,50]. For example, the detection of prey DNA in fecal samples has been shown to depend on consumer and prey combinations [50], differential digestion of soft-bodied and hard-bodied prey, variable gut transition times for different prey components and prey types [14], and choice of target sequence [51].

Chitinous exoskeleton of crustaceans is more likely to be retained in guts [52] in comparison to prey containing fewer hard parts, possibly because enzymatic attack is delayed when such structures are present in the guts [15,53]. In seahorses, as in many other fish species, digestion and gut transition times are variable and dependent on many factors [15]. In general, seahorses have a relatively fast gut passage time [54,55], hence gut contents can presumably provide information on only recently eaten prey. In the feeding trials, *Gammarus* sp. was detected in feces up to three days after feeding, indicating that the DNA metabarcoding permits the identification of prey eaten over several days. Furthermore, this method is sensitive for detecting secondary predation as observed, for example, in samples of seahorses fed on wild *P. aibuhitensis* in which genera *Urodasys* and *Alpheus* were also detected, in accordance with previous studies highlighting reliability of DNA-based methods for detecting indirect predation [56].

The DNA metabarcoding approach used here has several advantages in comparison with traditional methods. It is time and cost effective, while allowing the assessment of dietary composition of many individuals simultaneously, thus implying that the approach could be used to conduct analysis on large scales. Furthermore, this approach does not require strong taxonomic skills to identify the prey taxa, although the opinion of an expert taxonomist is crucial in these studies. However, it strongly depends on the reference databases, whose level of completeness represents one of the most critical issues in DNA-based diet analyses. Although building an exhaustive reference database on benthic invertebrates seems challenging due to their high diversity and hard taxonomic work, representative databases should be obtained at least at the family level to limit misidentifications or unrecognized sequences. Another possible limitation of this approach is related to the accuracy of the identification of the used barcode. Mitochondrial cox1 is a standard region used in animal DNA barcoding [57,58,59]. This marker has several advantages: its variation usually allows species-level discrimination and can be PCR amplified from most animals [60]. However, it has been acknowledged that primer binding sites within this protein-coding gene are not highly conserved [60], and indeed, many COI primers have highly variable amplification success even among members of the same group [61]. Consequently, some efforts must be made to increase the resolution power by using more than one primer pair to improve the amplification success.

In the present paper, the primary objective was to develop, test, and provide a protocol for DNA metabarcoding of fecal samples to study the diet of *H. guttulatus*, but valid for any other seahorse species. Any comprehensive characterization of the diet will require a larger sample size and year-round sampling.

Despite associated biological and technical challenges, the applied approach has many potential applications. Although it involves the capture and minimal handling, DNA metabarcoding allows for studying the diet using non-invasive sampling and does not require the sacrifice of animals, which is fundamental for sensitive species such as *H. guttulatus*. Comprehensive knowledge on the dietary composition of such species is an important step to plan conservation initiatives for local populations since shifts in diet could indicate the presence or absence of competitors, predators, or anthropogenic modifications of habitats. Additionally, the gained data may be also used to assess the composition of local communities, and the comparison of samples collected in different seasons may provide information not only on the diet variability, but also on community changes along the year. The small diversity of taxa exploited by the long-snouted seahorses between habitats supports the notion that this species is a specialist feeder [20]. Especially having in mind recently reported sharp decline of seahorse density at Taranto Mar Piccolo [62]; the use of taxa specific primers, coupled with a greater number of samples will allow the method to address significant ecological questions and could help at preserving this emblematic species.

## Figures and Tables

**Table 1:** Species observed in samples of seahorses fed on *Artemia franciscana*.

Taxa	FT1-AR	FT2-AR	FT3-AR	Mean	ST. DEV
*Nannochloropsis oceanica*	93.17%	88.93%	83.32%	88.47%	4.94%
*Artemia franciscana*	6.51%	7.65%	16.65%	10.27%	5.55%
Unassigned	0.23%	3.39%	0.02%		

**Table 2:** Species observed in samples of seahorses fed on *Gammarus aequicauda*.

Taxa	FT2-ANF	FT3-ANF	MEDIA	ST. DEV
*Nannochloropsis oceanica*	0.18%	21.4%	10.8%	14.9%
*Gammarus* sp.	98.4%	78.4%	88.4%	14.13%
Unassigned	1.44%	0.20%		

**Table 3:** Species observed in samples of seahorses fed on *Perinereis aibuhitensis*.

Taxa	FT2-PL	FT4-PL	Mean	ST. DEV
*Nannochloropsis oceanica*	0.01%	20.77%	10.39%	14.68%
*Perinereis aibuhitensis*	0.22%	14.44%	7.33%	10.05%
*Urodasys* sp.	33.70%	23.1%	28.4%	7.5%
*Alpheus* sp.	0.37%	1.99%	1.18%	1.14%
Unassigned	64.52%	39.29%		

**Table 4:** Species observed in samples of seahorses fed on *Palaemon elegans*.

Taxa	FT2-PA	FT3-PA	FT4-PA	Mean	ST. DEV
*Nannochloropsis oceanica*	8.79%	22.85%	8.88%	13.51%	8.09%
*Ophryotrocha labronica*	67.91%	7.57%	0	25.16%	37.22%
*Palaemon elegans*	13.14%	44.98%	30.86%	29.66%	15.95%
*Alpheus bellulus*	0	4.6%	0	1.53%	2.65%
Unassigned	1.00%	14.85%	6.18%	7.34%	6.99%

## Data Availability

Not applicable.

## Supplementary Materials

The following are available online at https://www.mdpi.com/article/10.3390/life11100998/s1, Figure S1: Boxplot of sequence counts: (i) PE reads: number of produced PE reads; (ii) Adaptor/Primer Trimming: number of PE reads retained after Illumina adapter and PCR primers trimming; (iii) Filtered: number of PE reads passing DADA2 quality filter; (v) Denoised F: number of denoised forward reads; (vi) Denoised R: number of denoised reverse reads; (vii) Assembled: number of merged PE reads; (vii) No Contaminants: number of retained sequences following removal of chimeric ASVs, human, *Hippocampus guttulatus* and prokaryotic sequences., Table S1: SIMPER analysis.
