# Supplementary material for: Evaluating the Efficiency of DNA Metabarcoding to Analyze the Diet of Hippocampus guttulatus (Teleostea: Syngnathidae)
Source: Life (Basel). 2021 Sep 22;11(10):998. doi: 10.3390/life11100998 (PMC8540156; doi:10.3390/life11100998)
Supplement: Supplementary file 1 [file life-11-00998-s001.zip › life-1382844-supplementary/life-1382844-figure S1.pdf]

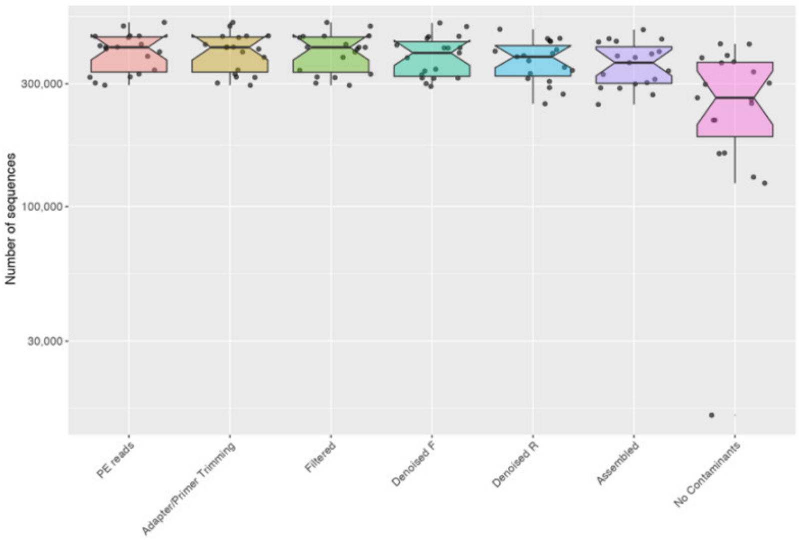

Figure S1. Boxplot of sequence counts: (i) PE reads: number of produced PE reads; (ii) Adaptor/Primer Trimming: number of PE reads retained after Illumina adapter and PCR primers trimming; (iii) Filtered: number of PE reads passing DADA2 quality filter; (v) Denoised F: number of denoised forward reads; (vi) Denoised R: number of denoised reverse reads; (vii) Assembled: number of merged PE reads; (vii) No Contaminants: number of retained sequences following removal of chimeric ASVs, human, Hippocampus guttulatus and prokaryotic sequences.
